# Application of Extreme Value Statistics to Corrosion

**DOI:** 10.6028/jres.099.030

**Published:** 1994

**Authors:** Toshio Shibata

**Affiliations:** Department of Materials Science and Processing, Faculty of Engineering, Osaka University, 2-1 Yamadaoka, Suita 565 JAPAN

**Keywords:** corrosion probability, failure time, Gumbel distribution, localized corrosion, maximum pit depth, MVLUE method, return period, Weibull distribution

## Abstract

Application of extreme value statistics to corrosion is reviewed. It is emphasized that the concept of corrosion probability is important for a quantitative evaluation of corrosion failure and its prediction, especially for localized corrosion. Extreme value statistics is quite useful for assessing the maximum pit depth and/or the minimum time for crack generation. The maximum pit depth depending on the surface area can be evaluated by using the Gumbel distribution with the concept of return period. A standardized procedure is proposed for estimating the maximum pit depth. Examples of corrosion failure analysis using extreme value statistics, which were reported mainly in Japan, are shown. Accumulated experiences suggest that an appropriate classification of data based on the corrosion mechanism is required before applying extreme value analysis.

## 1. Introduction

Development of extreme value statistics and its application to various fields, including corrosion, has been described by Gumbel in Ref. [1] and [2]. Evans is one of the pioneers of modern corrosion science, and first established the concept of corrosion probability [3, 4]. Eldredge [5] used extreme value statistics to obtain the maximum value of pit depth on an oil well tube wall as a function of tube surface area. Scott [6] found a logarithmic dependence of the maximum pit depth on surface area, and explained that dependence by referring to Trippet [7]. Aziz [8] and Eledredge [5] discussed almost all important points to be considered for the analysis of corrosion pit data and made use of the return period concept. This concept, originally introduced in the fields of hydrology or meteorology, is now used to obtain a size factor which makes it possible to estimate the maximum pit depth in a large surface area based on the distribution of a small number of pit depth data from the small surface area samples. In Japan, early review papers on corrosion probability and extreme value statistics by Masuko [9] and Shibata [10, 11] contributed to the study of the extreme value statistics as applied to corrosion problems. Ishikawa [12,13,14] and Imagawa [15,16,17] applied extreme value statistics to analyze engineering data. Kase [18, 19] reviewed Lieblein’s paper [20], introducing MVLUE (minimum variance linear unbiased estimator) method for estimating the distribution parameters, Lieblein had given the coefficient of MVLUE up to *N = 6*. Recently, Tsuge [21] had calculated the coefficients up to *N* = 45, and confirmed that the parameters estimated by the MVLUE method are unbiased and efficient and are consistent with values estimated by the method of moments or maximum likelihood when the sample size exceeds more than 20. The committee of Japan Society of Corrosion Engineering (JSCE) proposed a standard procedure [22] to estimate the maximum pit depth from the small sample size data by using the MVLUE method, and a computer program named EVAN [23] was developed. Recently Laycock et al. [24] reported that a generalized extreme value distribution is more convenient for corrosion depth analysis, because no preliminary assumption on the type of distribution is needed. An introductory book [25] by Kowaka et al. helped to differentiate extreme value analysis among corrosion workers in Japan. General background on extreme value statistics is provided by Ang and Tang [26], and Kinnison [27].

## 2. Application of the Extreme Value Analysis to Corrosion

In the early 1980s, meetings and symposia [28,29,30,31] were held in Japan for discussing the basic principles of extreme value statistics as well as difficulties and problems in their application to corrosion. In Table 1, several topics for which the Gumbel distribution is applied are listed. Table 2 includes cases analyzed using the Weibull distribution, including the exponential distribution. Before discussing case histories, the standard procedure [22] proposed by the committee is briefly explained; details are available elsewhere [22, 32].

### 2.1 The Gumbcl Distribution

The procedure is proposed mainly for analyzing pit depth distribution by using the Gumbel distribution and the return period in order to estimate the maximum depth of the larger surface area from which small area specimens are sampled. The Gumbel distribution is expressed as

F(x)=exp(−exp(−(x−λ)/α), (1)

where *F*(*x*) is the cumulative probability of pit depth, *x*, and λ and *α* are the location and scale parameters. The reduced variate, y,

y=(x−λ)/α (2)

is introduced, and then

y=−ln(−ln(F(y))) (3)

is used for constructing the Gumbel probability paper. Plotting position for the cumulative probability can be calculated simply by

F(y)=1−i/(1+N), (4)

where *i* is the *i* th of the ordered value, *x*, in descending order and *N* is the total number of sample. Plotting *y* as a function of *x* yields a best-fitting straight line; its slope provides 1/*α* and its intercept (at *y* = 0) yields A. Instead of this graphical estimation of the parameters, more reliable estimates of *α* and λ can be obtained by using the MVLUE (minimum variance unbiased estimator) method, the maximum likelihood and the method of moments. Among them the MVLUE method which is discussed by Lieblein [20] is found to be more efficient and unbiased for small size samples. The MVLUE estimator can be calculated by

$$\begin{array}{l} \lambda =\Sigma a_{i}\;(N,n)x_{i}\\ \alpha =\Sigma b_{i}\;(N,n)x_{i}, \end{array}$$ (5)

where *a_i_* (*N, n*) and *b_i_* (*N, n*) are weights for each sample depending on the sample size, *N*, and truncated number, *n*, which are tabulated in the table given by Tsuge [21, 23] up to *N=45*. The weights, *A, B, C*, of variance, *V*,

$$V=\alpha^{2}\;(A\;(N,n)y^{2}+B\;(N,n)y+C\;(N,n))$$ (6)

are also found in the table given by Tsuge [21, 23]. For the pit depth distribution, the return period, *T*, is defined as

T=S/s, (7)

where *S* is the surface object (e.g., a tank plate) to be examined and *s* is the area of the small specimens which are sampled randomly from the objective. The return period, *T*, is in effect a size factor. The mode, λ, of the pit depth distribution for the small specimen is simply obtained by the MVLUE estimators mentioned above, and the mode for the *T* times larger surface, *x*_max_ is given by

$$x_{\max}=\lambda +\alpha \ln\;(T).$$ (8)

The perforation probability, *P*, of the maximum pit through the wall thickness, *d*, is given by

P=1−exp(−exp(−(d−(λ+αln(T)/α)). (9)

Finally, the procedure [22] requires reporting the surface area of the object, *S*, with the small sample area, *s*, providing the return period, *T* (*= S/s*), and the number of samples, *N*, with data number, *n*, actually obtained. In addition, the original thickness of the plate, *d*, and the perforation probability, *P*, if needed are to be stated. The above procedure does not request to check a goodness of fit of the distribution obtained to the Gumbel distribution, but recommends to examine the fitness by the Kolmogorov-Smirnov or chi-square test if needed.

### 2.2 The Weibull Distribution

The third type for the smallest value called the Weibull distribution

$$F(t)=1-\exp\;(-{\left((t-\gamma )/\eta \right)}^{m})$$ (10)

can be fitted to the failure life distribution of stress corrosion cracking [33, 34] as shown in Table 2, where *y, η* and *m* are the location, scale and shape parameter, respectively. This third type of asymptotic distribution for the smallest value can be transformed to the first type for the largest value, that is, Eq. (1), by changing 1–*F*(*t*) to *F*(*z*) and by introducing the following reduced variate

X=ln(t−γ),z=(X−λ)/α. (11)

The same MVLUE method used for Eq. (1) can be utilized for parameter estimation, because the following relations exist between the parameters of both distributions;

λ=ln(η),α=1/m. (12)

The above unified procedure for estimating parameters of the Gumbel and Weibull distribution was coded in the computer program EVAN [23].

## 3. Examples

Several examples are provided to demonstrate the usefulness of extreme value statistics for analyzing corrosion problems.

### 3.1 Maximum Pit Depth of Oil Tank Plate

Through the 1960s and 1970s a number of oil tanks were built in Japan. In the late 1970s there occurred frequent oil leakages from tanks due to corrosion failure. Oil refinery or petrochemical industries were located along the seacoast and oil leakage caused serious environmental damages. In 1976, the fire service law was revised to enforce inspection of the thickness of the base and annular plates of oil tanks every time oil was evacuated. On these occasions extreme value analysis was applied and found to be a powerful tool for estimating the maximum pit depth. It is emphasized that data for the base plate and the annular plate should be considered separately because they are characterized by different corrosion damage and mechanisms.

The law requests that plate thickness has to be measured at the corners of every 10 cm square on the whole surface of the plate. This inspection procedure contributed greatly to reducing corrosion leakage, but was time-consuming and costly. The extreme value analysis was then studied intensively in this field [35] [36]. Pit depth distribution sampled from the whole base plate was found to obey the Poisson distribution.

**Fig. 1:**
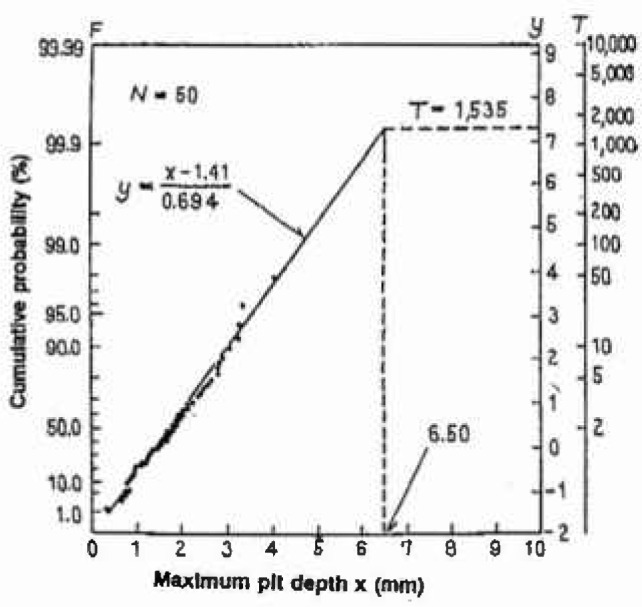
The Gumbel plot of the maximum pits on the bottom plate of the oil tank.

**Fig. 2:**
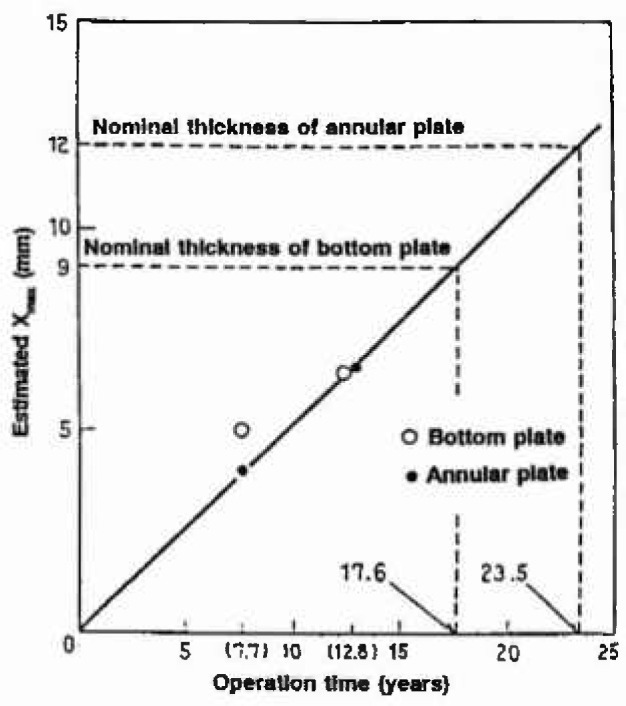
Estimated depth of the maximum pit on the whole surface as a function of operation years, and prediction of failure life for the bottom and annular plate.

Araki et al. [36] found that the largest value from each small square (*s* = 1 m^2^) being randomly sampled obeys the Gumbel distribution as shown in Fig. 1 [36]. The slope and intersect of the line (*α* = 0.694 and *λ* =1.41) were estimated by the MVLUE estimators of Eq. (5). In this case, the surface area of the base plate, *S*, was 1535 m^2^ and the return period or size factor was calculated to be *T = S/s* = 1535. The maximum depth, *x*_max_, was calculated by Eq. (8):

$$x_{\max}=1.41+0.694\times \ln\;(1535)=6.50\;\mathrm{mm}$$

which is shown also in Fig. 1. These data were obtained for a base plate which was exposed for 12.6 years. Data for both annular and base plate exposed for 7.7 years were plotted in Fig. 2 [36], from which the first leak due to the maximum pit is to be expected after 17.6 years for the base plate and 23.5 years for the annular plate, respectively. The effect of *N, s*, on the estimates was examined and it was concluded that the MVLUE method is optimal for *N* <20 and the maximum likelihood method is reliable for *N* > 20.

### 3.2 Rupture of Heat Exchanger Tubes of the Boiler

**Fig. 3:**
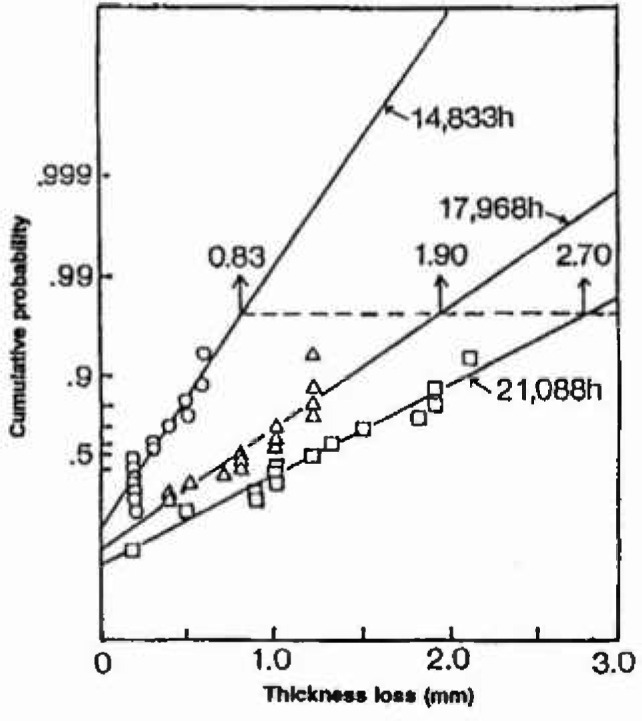
The Gumbel plots of the maximum thickness loss of boiler tubes used for different operation times.

**Fig. 4:**
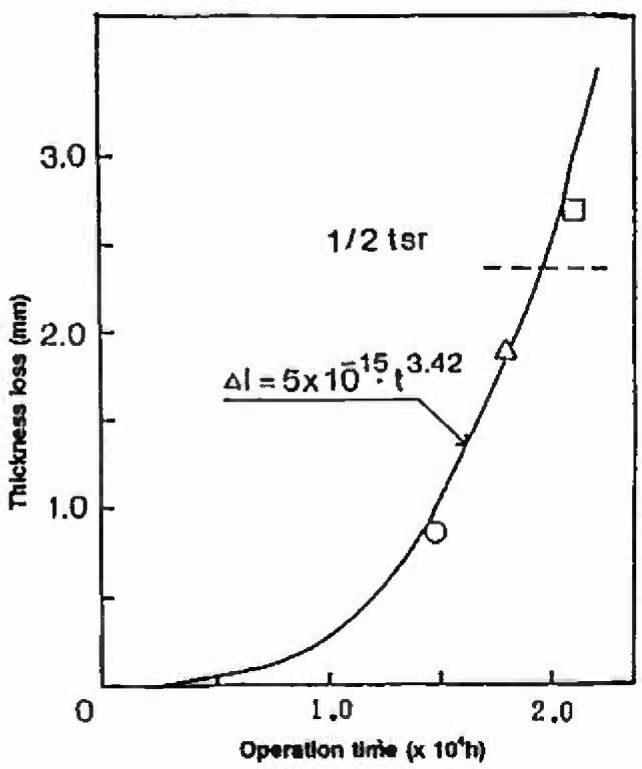
Thickness loss of boiler lubes as a function of operation time and estimation of rupture time.

Super heater and economizer tubes of boilers are exposed to high temperature gases with salt deposits which cause severe corrosion attack. Corrosion attack is not uniform, but localized at several sites, wall thinning at the localized site resulting in burst. Regular inspection is needed to predict time for replacement of the tube before burst. All tubes have to be examined for predicting exact time with high confidence, but cost of inspection being high, that Fukuda et al. [37] introduced the use of extreme value analysis to supplement the inspection of a small number of tubes. In Fig. 3, the largest values of wall thinning observed for 14 tubes are plotted on Gumbel probability paper. The distribution of wall thinning at every inspection time is seen to obey the Gumbel distribution and the maximum thickness determined by the return period (40 tubes) increases with operation time as shown in Fig. 4. A criterion for a proper replacement time has been proposed, which requires replacement when the wall thickness reaches half of the design thickness, *t_sγ_*. Risk of burst could be avoided by estimating the depth and noting the proposed criterion.

### 3.3 The Pit Depth Distribution of Steel Piles in Sea Water

**Fig. 5:**
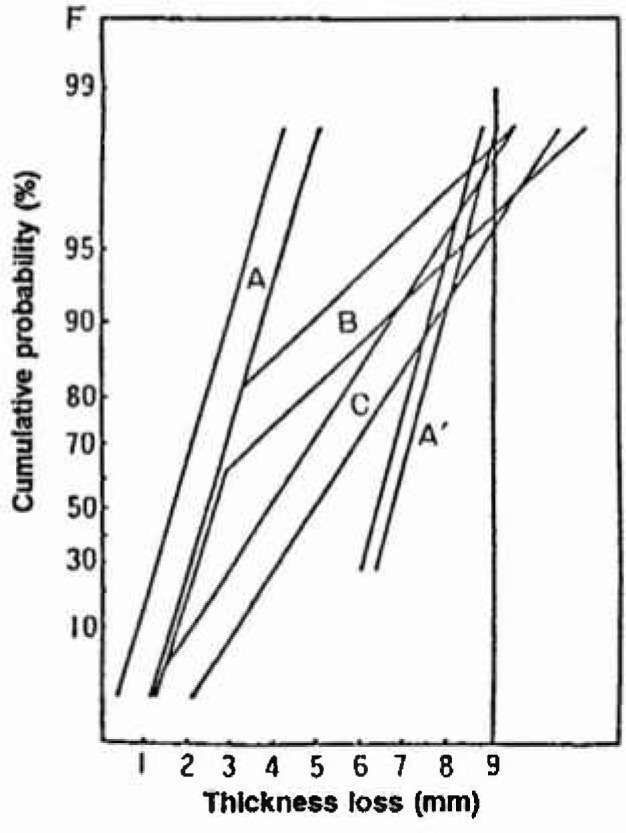
Various types of the distribution observed for steel piles and pipes exposed in sea water.

Since the 1970s, steel pipes and piles have been used extensively in Japan for harbor construction, because lead time for construction could be reduced compared with using concrete. Recently, corrosion of steel pipes and piles was found to cause the collapse of harbor structures. Then corrosion damage of steel structures exposed in sea water has been inspected and analyzed by using extreme value analysis. Itoh et al. [38] reported that three different types of depth distribution were found for steel piles and plates depending on exposure time and exposure location such as water level and deep sea (Fig. 5). The type A distribution which exhibits a nearly straight line, was found for uniform corrosion loss, its mean value being below 1.0–1.2 mm thickness. The type C distribution obeying the Gumbel distribution was observed for heavily localized specimens. The type B distribution is a mixed type of A and C distributions. The estimated depth using the return period was consistent with observations.

### 3.4 Classification of Data Based on Corrosion Knowledge

In any of the cases mentioned earlier, measured sets of data is fitted by two or three distributions and must be separated from each other before the analysis in order to obtain the maximum value. Imagawa et al. [15,16,17,39] presented many cases which require classification of data. For example, data for the heat exchanger tubes had to be classified into the inlet and outlet side samples because corrosion form and its degree of damage were different at the two locations owing to exposure to different temperatures. For the oil tank, Imagawa observed that more deep pits were formed on the welding line compared with other parts. He obtained the different estimated value of the pit depth for each classified sample. At the present time, the classification was done on corrosion knowledge and experience, but it is required to establish a procedure based on a common criterion.

### 3.5 Crack Depth Distribution of Stress Corrosion Cracking

Stress corrosion cracking is one of the most dangerous corrosion failure and shows random occurrence which is a very specific and common feature of materials fracture. The Weibull distribution has been known to be quite useful to analyze the distribution of fracture strength of various materials [40] and also has been found to be applicable for analyzing failure life distribution due to stress corrosion cracking [33, 34].

**Fig. 6:**
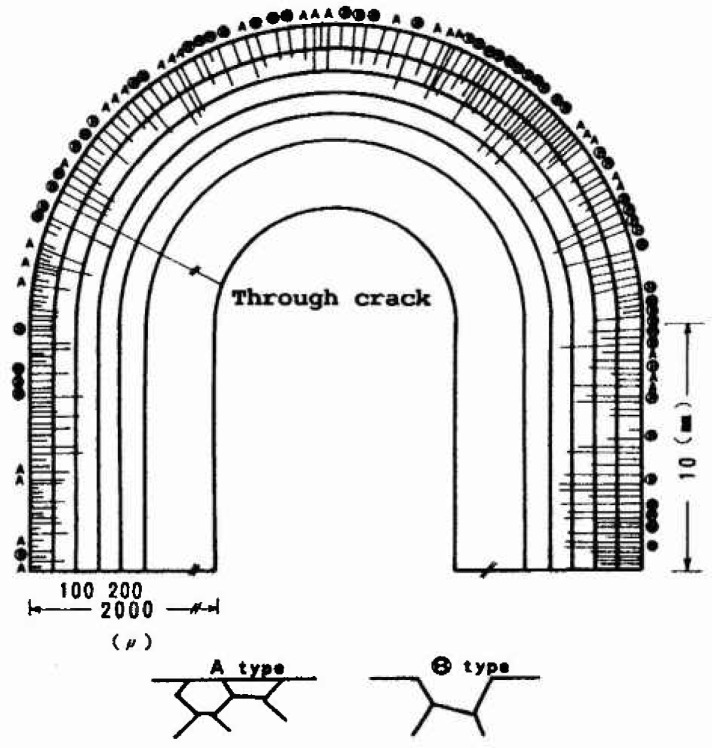
The distribution of intergranular corrosion attacks and cracks observed for sensitized type 304 stainless steel exposed to the BWR simulated water (DO 8 ppm, 250 °C).

**Fig. 7:**
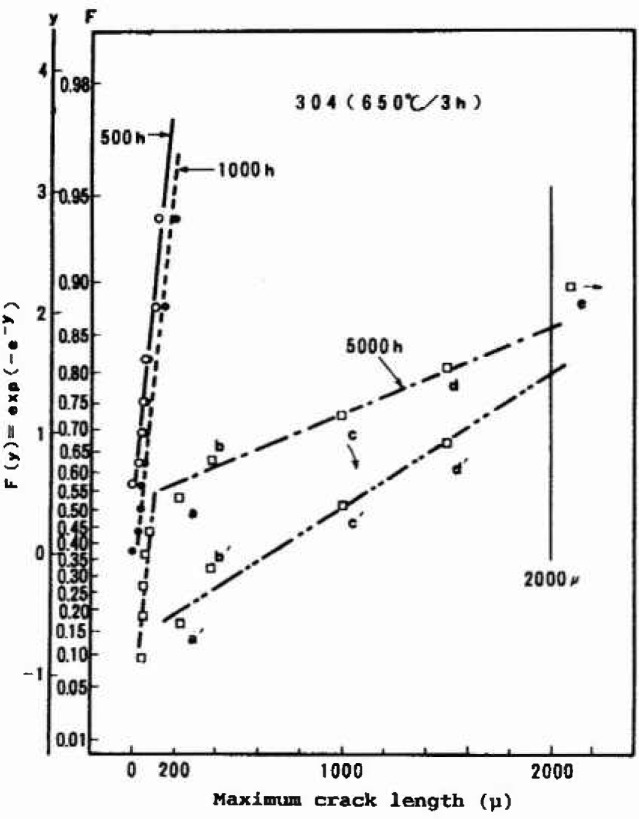
Distributions changing with exposure time, the initial distribution corresponding to intergranular corrosion and the second to intergranular cracking.

An interesting application of the Gumbel distribution for analyzing the crack depth distribution has been reported by Tsuge [41]. The laboratory experiment for evaluating the susceptibility of stress corrosion cracking of Type 304 stainless steel was done by using a bent specimen of u-shape. Bending gives stress to the specimen and the environmental condition of high pressure water causes many cracks, which can be revealed by sectioning the specimen after the test as shown in Fig. 6. Distribution of the crack depth plotted in the Gumbel probability paper showed two lines with an inflection point as can be seen in Fig. 7. This inflection point was found to correspond just to a depth for initiating the intergranular crack. Thus the initiation of the intergranular crack growth could be separated from the initial process of purely chemical intergranular corrosion.

### 3.6 Estimation of the Maximum Segregation of Impurities in Steel

Continuous casting of steel is one of the innovative technologies achieved by the steel industry. Segregation and its band which are formed during solidification at the center of slab remain after rolling and work as initiation sites for fracture phenomena such as lamellar tear and hydrogen induced cracking (HIC). The maximum amount of segregation was found to be related to the above fracture phenomena, so that extreme value analysis was applied for estimating the maximum amount of segregation in steel plate from small area samples [42], the concentration of impurities being measured by using EPMA. The maximum amount of segregation thus determined can be used to predict the susceptibility to lamellar tear. It should be emphasized that the statistical procedure for the chemical analysis is mainly concerned with the mean and standard deviation which assesses the reliability of the measurement, but not with extreme values. In recent years, highly sensitive analytical methods have been developed, but it is not clear how to correlate the data of small area samples to that of the total or bulk specimen. The ratio of the analytical area to the bulk specimen reaches almost to 10^−9^, and extreme value statistics is expected to be useful.

### 3.7 Non-Destructive Methods With the Extreme Value Analysis

Various types of nondestructive methods are used for inspecting and examining corrosion damage in order to prevent failure. High sensitivity and resolution in time or in space are required for the measurement. In addition, a computer-aided operation becomes popular, because huge amounts of data must be evaluated. For the heat exchanger, a thousand tubes must be checked and the number of data easily exceeds 10^5^. An eddy current sensor [43] and ultrasonic sensor probe [44] to steel tubes, and an impedance sensor probe [45] for coating tubes have been developed with the data logger and the extreme value analysis software.

## 4. Discussion

The size effect on the maximum pit depth is found to be estimated with confidence by introducing the concept of the return period. Theoretical bases of the procedure have been provided by extreme value theory [1]. Our experience shows that the pit depth distribution obeys the normal or exponential distribution, which belong to the exponential distribution family. Thus the maximum values of pit depth extracted from the exponential family distribution may reasonably be expected to obey the Gumbel distribution. Thus the size effect could be rationally predicted by using the concept of return period.

Evans [46] pointed out, however, that some cases as observed by Wormwell et al. [47] does not obey a normal or exponential type distribution, but that the tail of the distribution is limited at a certain depth. Evans emphasized that such a limit is reasonable for the case of anodic reaction control situation and this limited depth gives a rough indication of the greatest pit depth to be expected on a much larger area. Evans, however, did not check another possibility using the type HI distribution which has an upper limit. Recently Laycock et al. [24] discussed that usefulness of the generalized extreme value (GEV) distribution:

$$F(x)=\exp\;(-{\left(1-k(x-u)/\alpha \right)}^{1/k}\;kx<\;=\alpha +uk,$$ (13)

because the distribution subsumes all three types with the sign of a shape parameter, *k*. When *k* is zero, negative or positive, the distribution changes to type I, type II, and type HI, respectively. They found that the pit distribution on stainless steels in acidified chloride solution fits the GEV distribution with *k* positive, indicating that the type III for the largest value could be fitted. The type III distribution has a bound or a limit with increasing area, as suggested by Evans.

What sample size, or what size of specimen area should be used are questions from non-specialists in statistics. For this question, we proposed a procedure or criterion for choosing *s, N* and *T* based on the variance given by Eq. (6). The surface area, *S*, of the object is given, and the sampling area, *s*, is selected so as to include at least one pit. Then *T*(*= S/s*) is obtained. Accumulated data of the parameters of *α* and *λ* suggest [32] that the ratio of *α*/*λ* for localized corrosion is below, or not much larger than, 0.3. Kinnison [48] states that the asymptotic theory predicts a constant ratio of 0.313 for all extreme value distributions. Then it can be assumed that the ratio *α*/*λ*, is 0.3. If we wish to control variance within (*λ*/3)^2^, the following relation can be deduced from Eq. (6)

$${\left(\lambda /\alpha 3\right)}^{2}=A(N,n)y^{2}+B(N,n)y+C.$$ (14)

**Fig. 8:**
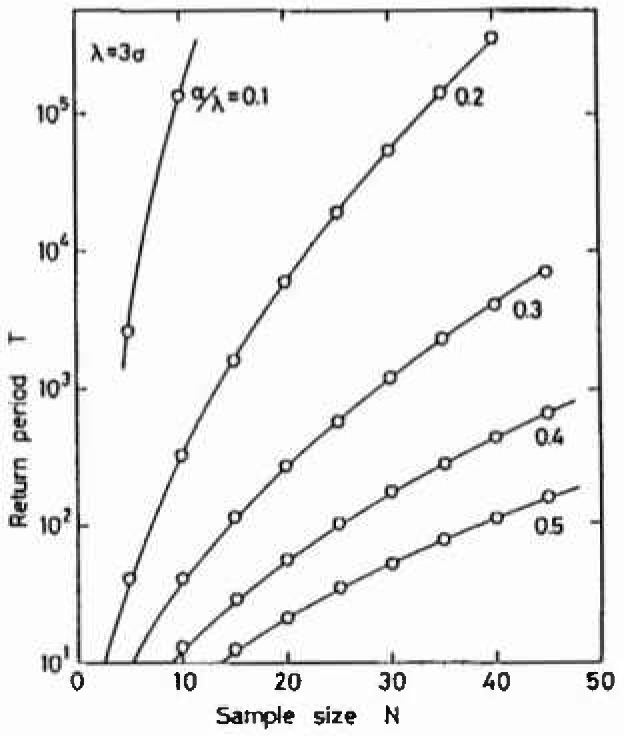
Calculated curves of the optimum condition to choose *T* and *N* at various value of *α*/*λ*.

Equation (14) can be solved for *y* or *T* as a function of *N* and *α*/*λ*, as plotted in Fig. 8. When the ratio of *α*/*λ* can be equated to 0.3 as discussed before, a suitable number of samples can be found for a given return period, *T*. From this figure, the required size of samples is *N* = 30 for *T* = 1200, or *N* = 20 for *T* = 274 and so on. This figure is approximately the same as what was observed empirically.

## 5. Conclusions

Extreme value statistics has been found to be a powerful tool for estimating the maximum value of localized corrosion depending on the surface area. Accumulation of data and experience, however, reveals that statistics is less important than corrosion experience and knowledge for obtaining a reasonable estimation; measured data must be classified based on the form of corrosion damage and its degree before the analysis. Properly classified data is found to provide a very reasonable value. Nondestructive methods for measuring wall thickness with various types of sensors, combined with extreme value analysis, have been developed in recent years.

## Figures and Tables

**Table 1:** Examples of the extreme value analysis for corrosion using the Gumbel distribution

Example	Ref.
1. Life prediction of super heater tubes of the power plant	[49]
2. Application of the extreme value analysis to heating tubes of the boiler	[37]
3. Estimation of the maximum amount of impurity segregation in steel	[42]
4. Failure life estimation of SCC for Ni base alloys	[41]
5. Extreme value analysis of the corrosion depth of the oil tank plate	[35]
	[36]
	[50]
6. Life prediction of heat exchanger tubes	[51]
7. Eddy current examination system for heal exchanger tubes with the extreme value analysis	[43]
8. Extreme value analysis of pitting corrosion of heat exchanger tubes	[52]
9. Methods for the parameter estimation of the pit distribution in plants	[16]
10. Ultrasonic method for heat exchanger tubes with the extreme value analysis	[44]
11. Maintenance system for coated heat exchanger tubes	[45]
12. Corrosion of steels in sea water	[53]
13. Analysis of perforation of zinc plating steels by extreme value statistics	[54]
14. Fatigue crack behavior of high strength steel in artificial sea water	[55]

**Table 2:** Examples of the extreme value analysis for corrosion using the Weibull (exponential) distribution

Example	Ref.
1. Failure life analysis of stress corrosion cracking of stainless steel heat exchanger tubes	[56]
2. Failure life distribution of stainless steels in high temperature and high pressure water	[57]
3. Effect of CaCl_2_ concentration on SCC life time distributions of stainless steels	[58]
4. Evaluation of SCC failure life of stainless steel in high temperature water	[59]

## References

[1] Gumbel EJ (1958). Statistics of Extremes.

[2] Gumbel EJ (1954). Statistical Theory of Extreme Values and Some Practical Applications.

[3] Mears RB, Evans UR (1935). The “Probability” of Corrosion. Trans Faraday Soc.

[4] Evans UR (1960). The Corrosion and Oxidation of Metals: Scientific Principles and Practical Applications.

[5] Eldredge GG (1957). Corrosion.

[6] Scott GN (1934). Proc A Petroleum Inst.

[7] Trippet LHC (1925). Biometrika.

[8] Aziz PM (1956). Corrosion.

[9] Masuko N (1972). Boshoku Gijutsu.

[10] Shibata T (1978). Boshoku Gijutsu.

[11] Shibata T (1980). Kinzoku Hyomen Gijutsu.

[12] Ishikawa Y (1979). Boshoku Gijutsu.

[13] Ishikawa Y, Ozaki T, Hosaka N, Nishida O (1981). J Soc Mater Sci Jpn.

[14] Ishikawa Y, Ozaki T, Hosaka N, Nishida O (1982). Trans 1SIJ.

[15] Imagawa H, Matsuno K (1982). Boshoku Gijutsu.

[16] Imagawa H, Matsuno K (1987). J Soc Mater Sci Jpn.

[17] Imagawa H, Matsuno K (1989). J Soc Mater Sci Jpn.

[18] Kase S (1982). Boshoku Gijutsu.

[19] Kase S (1983). How to Analyze Reliability Data—Double Exponential Distribution.

[20] Lieblein J (1954). A New Method of Analyzing Extreme-Value Data, National Advisory Committee For Aeronautics.

[21] Tsuge H (1987). J Soc Mater Sci Jpn.

[22] JSCE 60-1 Technical Committee, Working Group (1988). Corrosion Eng.

[23] JSCE 60-1 Technical Committee, Working Group (1989). Computer program “EVAN”.

[24] Laycock PJ, Cottis RA, Scarf PA (1990). J Electrochcm Soc.

[25] Kowaka M, Tsuge H, Akashi M, Masamura K, Ishimoto H (1984). An Introduction to the Life Prediction of Plant Materials—Application of Extreme Value Statistical Methods for Corrosion Analysis.

[26] Ang AH-S, Tang WH (1984). Probabilistic Concepts in Engineering Planning and Design, Vol II — Decision, Risk, and Reliability.

[27] Kinnisun RR (1985). Applied Extreme Value Statistics.

[28] 41st Corrosion and Protection Symposium (1982). Life Prediction by Extreme Value Statistics.

[29] 51st Corrosion and Protection Symposium (1983). Life Prediction of Plant Materials.

[30] 58th Corrosion and Protection Symposium (1984). Life Prediction of Plant Materials—Application of Extreme Value Statistics to Corrosion.

[31] 73rd Corrosion and Protection Symposium (1988). Life Prediction of Plant Materials—Experience and Practice of the Analysis.

[32] Shibata T (1991). ISIJ International.

[33] Shibata T, Hine F, Komai K, Yamakawa K (1988). Localized Corrosion.

[34] Shibata T, Parkins RN Proc Life Prediction of Corrodible Structures.

[35] Shibata T, Okamoto K (1981). Boshoku Gijutsu.

[36] Araki R, Miura A, Sakai M, Yokoyama J, Yokoya S (1986). Atsuryoku Gijutsu.

[b37-jresv99n4p327_a1b] 37Y. Fukuda, H. Kawasaki, and M. Seki, in Ref. [30], p. 24.

[38] Itoh S, Murata T, Okada H (1984). Poc Inter Congr Metallic Corrosion.

[39] Imagawa H (1987). Haikan Gijutsu (Piping Engineering).

[40] Weibull W (1951). J Applied Mechanics, ASME.

[b41-jresv99n4p327_a1b] 41H. Tsuge, in Ref. [29], 16–21 (in Japanese.)

[b42-jresv99n4p327_a1b] 42H. Ogawa, in Ret [30], 31–36.

[43] Anzai T, Yamamoto H, Wakebe K (1990). Bosei Kanri.

[44] Kimura M (1987). Haikan Gijutsu.

[45] Kurusu S, Sato T (1987). Haikan Gijutsu.

[46] Evans UR (1960). The Corrosion and Oxidation of Metals: Scientific Principles and Practical Applications.

[47] Wormwell F, Butler G, Beynon JG (1957). Trans Inst Marine Eng.

[48] Kinnison RR (1985). Applied Extreme Value Statistics.

[b49-jresv99n4p327_a1b] 49S. Kobayashi, in Ref. [30], pp. 8–13.

[b50-jresv99n4p327_a1b] 50K. Malsuno and H. Imagawa, in Ref. [31], pp. 8–15.

[b51-jresv99n4p327_a1b] 51H. Ishimoto, in Ref. [29], pp. 56–62.

[52] Takasaki S, Kontani T (1987). J Soc Mater Sci Jpn.

[53] Blekkenhorst F, Frerrari GM, Van Der Wecken CJ, Ijsscling FP (1986). Br Corros J.

[54] Sato H, Shimogori K, Nishimoto H, Miki K, Ikeda K, Iwai M, Sakai H, Nomura S (1986). Testu-to-Hagane.

[55] Komai K, Minoshima K, Kim K (1987). J Soc Mater Sci.

[56] Committee of Materials for Chemical Industry Plants (1984). Society of Chemical Engineering, Japan.

[57] Clarke WL, Gordon GM (1973). Corrosion.

[58] Shibata T, Nakata J, Fujimoto S, Parkins RN Life Prediction of Corrodible Structures.

[59] Akashi M, Hine F, Komai K, Yamakawa K (1988). Localized Corrosion.

